# Central Retinal Artery Occlusion with Concomitant Intracranial Hemorrhage Secondary to *Streptococcus Gordonii* Endocarditis

**DOI:** 10.1155/2023/9268480

**Published:** 2023-05-11

**Authors:** Harshvardhan Chawla, Jonah S. Goldblatt, John E. Morgan, Bruce A. Barron, Aravinda K. Rao, Maria A. Reinoso

**Affiliations:** Department of Ophthalmology, Louisiana State University Health Sciences Center, 533 Bolivar Street, Suite 459, New Orleans, LA 70112, USA

## Abstract

**Purpose:**

To report a case of central retinal artery occlusion (CRAO) associated with subacute *Streptococcus gordonii* endocarditis secondary to a dental infection. *Observations*. A 27-year-old male presented with acute monocular vision loss in the setting of a stroke and seizure. A fundus exam revealed macular whitening and a cherry-red spot. Edema of the inner retinal layers was confirmed on macular optical coherence tomography, consistent with CRAO. Initial imaging (carotid Doppler, EKG, and transthoracic echocardiography) and a comprehensive laboratory workup did not reveal an etiology for the stroke or vision loss. Brain magnetic resonance imaging showed T1 hyperintensity with surrounding edema, which prompted a workup for possible septic emboli versus occult malignancy. Subsequent blood cultures led to the detection and diagnosis of *Streptococcus gordonii* endocarditis. It was subsequently revealed that the patient had self-extracted his molar two months prior to the onset of symptoms.

**Conclusions:**

Endocarditis has been associated with Roth spots and inflammatory findings in the posterior segment. However, CRAO caused by vegetal septic embolism is rare. To our knowledge, this represents the first reported case of endocarditic CRAO with *Streptococcus gordonii* confirmed as the causative microbe. Retinal vascular occlusion in a young patient with no distinct risk factors should prompt a comprehensive dental history and infectious workup, with consideration given to early transesophageal echocardiography.

## 1. Introduction

Central retinal artery occlusion (CRAO) is a severe ocular complication leading to irreversible vision loss [1]. Characterized by acute painless monocular vision loss, this condition presents with retinal whitening and a foveal cherry red spot [2]. In the acute stage, arteriolar “boxcarring” indicative of poor blood flow may be present. Macular optical coherence tomography (OCT) demonstrates a thickened, edematous inner retina with hyperreflectivity [3]. This is followed by long-term atrophy of the neurosensory retina [3].

The majority of CRAO occur secondary to vascular embolism [1]. These emboli have been shown to consist of cholesterol (74%), platelet fibrin (15.5%), and calcium (10.5%) [1, 4]. The former two are associated with carotid stenosis, while the latter has been linked to cardiac valve disease. CRAO secondary to giant cell arteritis (GCA) comprises approximately 5% of cases and requires emergent intravenous (IV) steroid therapy to prevent bilateral blindness [1]. Less common etiologies include vasculitis, trauma, sickle cell disease, hypercoagulable states, oral contraceptive use, and radiation exposure [5]. The visual prognosis is typically poor due to the onset of ischemic damage within three hours of the onset of symptoms, and patients are unlikely to receive stroke interventions within this timeframe [6].

CRAO secondary to endocarditis is a rarely reported phenomenon. This results from valvular vegetation embolizing into the systemic vasculature, with the causative microbes often unknown due to negative blood cultures at the time of diagnosis [2, 5–7]. Although uncommon, this represents a key mechanism to consider in cases of atypical CRAO given the potentially life-threatening sequelae. We present a novel case of CRAO secondary to *Streptococcus gordonii* endocarditis, caused by a dental infection two months prior to the onset of visual symptoms.

## 2. Case Presentation

A 27-year-old male presented to the emergency department following a syncopal event. He reported a severe headache and total loss of vision in the left eye, both of which had worsened over the preceding four days. His medical history included migraines, hypertension, prediabetes, Graves' disease, and pericarditis. While pending discharge after IV fluid administration, the patient experienced a tonic-clonic seizure. Computed tomography (CT) revealed a left-sided parietal intraparenchymal hemorrhage and an occipital subarachnoid hemorrhage (SAH). He was loaded with levetiracetam for seizure prophylaxis and transferred to the University Medical Center New Orleans (UMCNO) for neurosurgical evaluation. The patient underwent a cerebral angiogram, which revealed no structural etiology (i.e., arteriovenous malformation/fistula or mycotic aneurysm). He was subsequently admitted for neurologic checks and serial head CT scans.

**Figure 1:**
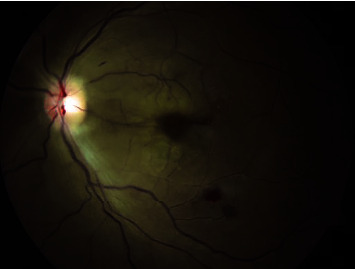
Fundus photograph, left eye. Macular whitening with a foveal cherry-red spot can be observed in the affected eye. Also present are two foci of intraretinal hemorrhage and two Roth spots in the macular periphery.

**Figure 2:**
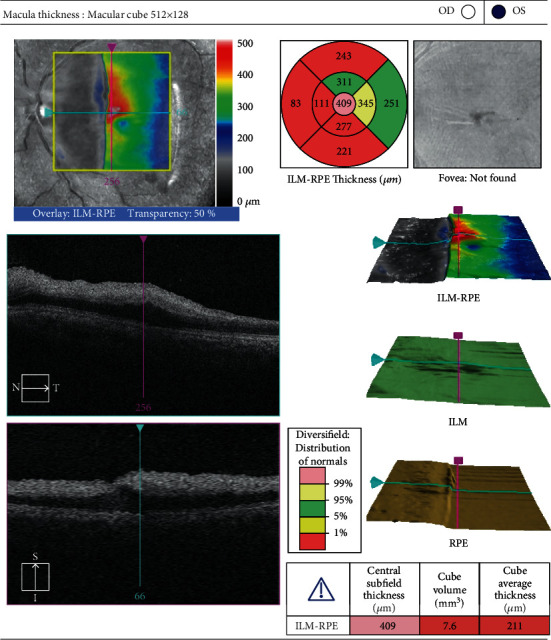
Macular optical coherence tomography. Imaging demonstrates a significant increase in central subfield thickness with obliteration of the foveal contour. Edema and hyperreflectivity obscure the distinction between the inner retinal layers.

The ophthalmology service was consulted the following day for acute monocular vision loss. The visual symptoms had manifested four days prior to presentation with increasing blurriness. The patient denied any preceding trauma or IV drug use. He denied any photopsia, myodesopsias, or “curtain” phenomenon. There was no family history of ocular or cerebrovascular disease. On exam, visual acuity (VA) was 20/20 in the right eye and counting fingers in the left eye. There was a 2+ afferent pupillary defect and total visual field deficit present in the left eye. Intraocular pressures were normal in both eyes. The anterior segment exam was unremarkable. A dilated fundus exam was normal in the right eye. There was macular whitening with a foveal cherry-red spot in the left eye, with two foci of intraretinal hemorrhage, and two Roth spots in the macular periphery (Figure 1). Retinal vessels were of normal caliber with no visible emboli or boxcar segmentation. Macular OCT demonstrated hyper-reflectivity of the inner retina with edema and a loss of definition between layers (Figure 2).

The ophthalmic differential diagnosis included CRAO, GCA, sphingolipidoses (Tay-Sachs disease, Niemann-Pick disease, Sandhoff disease, Fabry disease, and Gaucher's disease), and sialidosis. While CRP and ESR were both elevated (5.3 mg/dL and 50.0 mm/hour, respectively), GCA was considered unlikely given the patient's young age. Sphingolipidoses and sialidosis were also deemed unlikely in the absence of systemic abnormalities. A stroke workup was recommended based on a provisional diagnosis of CRAO.

The sequence of diagnostic testing (with time since the initial presentation) is listed in Table 1. Carotid Doppler did not demonstrate stenosis, and the EKG was unremarkable. A transthoracic echocardiogram (TTE) demonstrated only mild mitral regurgitation. Given the patient's age, labs were obtained to exclude alternative causes of vascular occlusion. Rheumatoid factor, antinuclear antigen, ANCA, RPR, FTA-ABS, and HIV were negative. Serum protein electrophoresis was compatible with a subacute inflammatory reaction (hypoalbuminemia with increased alpha-1 fraction and polyclonal gammopathy). Hemoglobin electrophoresis was unremarkable. Lipid levels were normal with the exception of a low HDL cholesterol. The INR was slightly elevated (1.3). Prothrombin time (PTT) was normal.

**Figure 3:**
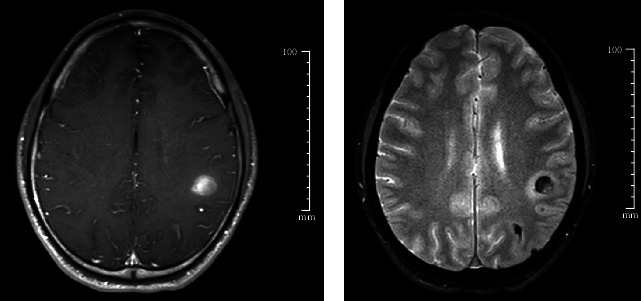
Brain magnetic resonance imaging with contrast. A circumscribed lesion with ring enhancement and hyperintensity on the T1-weighted image (a) with surrounding edema visible on the T2-weighted image (b).

Following these largely unremarkable results, the patient underwent a brain MRI three days after the initial CT scan. This demonstrated a circumscribed lesion of the parietal lobe with T1 hyperintensity and ring enhancement and T2 surrounding edema (Figure 3). Of note, the lesion was situated in the same region as the intraparenchymal hemorrhage identified on the initial CT scan. This prompted a secondary workup to exclude (1) septic embolism or (2) hemorrhagic malignancy (either primary or metastatic, with the latter deemed more likely). Blood cultures were obtained the same day.

The following day (4 days from presentation), the blood cultures grew Gram-positive cocci that were later speciated as *Streptococcus gordonii*. Antibiotic therapy consisting of intravenous vancomycin and ceftriaxone (deescalated to ceftriaxone alone following speciation) was begun immediately. On further questioning, the patient admitted to having self-extracted his molar two months prior. He then disclosed that he had experienced chills and rigors leading up to the onset of his visual and neurologic symptoms. A subsequent transesophageal echocardiogram (TEE) revealed mitral valve leaflet vegetations which had not been detected on the TTE performed 8 days earlier. Repeat blood cultures after 48 hours of antibiotic treatment were negative. A repeat head CT demonstrated the expected evolution of the previous hemorrhages but no new lesions or hemorrhages. The patient was therefore discharged twelve days from presentation/admission on outpatient IV ceftriaxone for six weeks (total antibiotic duration = 50 days). At five months, VA in the affected eye was stable at counting fingers.

## 3. Discussion

*Streptococcus gordonii* is a commensal gram-positive coccus found in the skin, oral cavity, upper respiratory tract, and gastrointestinal tract [8]. Typically nonpathogenic, this bacterium is most commonly associated with biofilm formation of the surface of teeth [8]. However, it has also been implicated in periodontal disease, septic arthritis, and spontaneous bacterial peritonitis. Infective endocarditis secondary to *Streptococcus gordonii* is exceedingly rare, and this represents the first reported case of endocarditic CRAO with *Streptococcus gordonii* confirmed as the causative microbe.

Infective endocarditis is characterized by the seeding of the cardiac valve endothelium by bacteria present in the systemic vasculature. Vegetations are formed by platelet-fibrin aggregation around these bacteria and can lead to septic embolism with a mortality rate of 10-30% [9]. Common manifestations of these emboli include splinter hemorrhages and Janeway lesions (nontender hemorrhagic macular spots on the palms and soles secondary to septic emboli microabscesses and resultant small vessel thrombosis). However, CRAO secondary to endocarditis is uncommon as 90% of central nervous system emboli ultimately travel to the middle cerebral artery [6]. Of note, the primary parietal hemorrhage in our patient was accompanied by a small occipital SAH. This likely represents a rare manifestation of endocarditis, for which the exact mechanism remains unclear [10, 11]. Proposed mechanisms for this phenomenon (distinct from that of mycotic aneurysm formation) center on septic erosion of vessel walls following the deposition of embolic matter, leading to eventual spontaneous rupture [10, 11].

From a management standpoint, there is no universally accepted treatment to reverse retinal damage caused by CRAO. Proposed methods include topical intraocular pressure-lowering agents, anterior chamber paracentesis, IV hyperosmotic solutions, and ocular massage [6]. Despite this limitation, prompt identification of the underlying cause is critical given the potentially life-threatening sequelae of infectious endocarditis as an underlying etiology. Patients with an atypical demographic (i.e., young age) and/or risk profile for CRAO should be specifically asked about dental symptoms and undergo an early infectious workup. Furthermore, this case was notable in that TEE revealed valve vegetation missed by the preceding TTE. This underscores the comparative sensitivity of TTE (44-63%) versus TEE (91-100%) for detection of infectious endocarditis in the existing cardiology literature [12]. Given this difference, it would be prudent to confirm a negative TTE with TEE in similar clinical situations.

Our patient underwent a comprehensive stroke workup incorporating a carotid ultrasound, an EKG, echocardiography, and an extensive lab panel [1]. However, it was ultimately in conjunction with the neurosurgery, neurology, and infectious disease consult services that diagnostics were widened to rule out septic emboli and occult malignancy. As emphasized by Serras-Pereira et al., such cases demonstrate the importance of multidisciplinary collaboration [2]. In summary, CRAO in an atypical patient should raise suspicion for endocarditis (particularly from opportunistic oral flora), with consideration given to early TEE imaging.

## Figures and Tables

**Table 1:** Clinical Timeline.

Diagnostic modality	Days from presentation	Results
CT head	0	Parietal intraparenchymal hemorrhage, occipital subarachnoid hemorrhage
CT angiogram, head and neck	0	Unremarkable, no arterial abnormality
Carotid ultrasound	3	Unremarkable, anterograde vertebral artery flow
Transthoracic echocardiogram	3	LVEF of 55-60%, no left to right shunt, mild mitral regurgitation
MRI brain	3	Parietal intraparenchymal lesion (T1 enhancement, T2 surrounding edema)
Blood cultures (collected)	3	—
Blood cultures (resulted)	4	Gram positive cocci
Commenced intravenous antibiotics	4	—
Blood cultures (speciated)	6	*Streptococcus gordonii*
Transesophageal echocardiogram	11	Anterior mitral valve vegetation
Discharged with PICC line	12	—

CT: computed tomography; MRI: magnetic resonance imaging; PICC: peripheral intravenous central catheter, LVEF: left ventricular ejection fraction.

## Data Availability

The relevant data for this case is included in the report. Additional data may be requested by contacting the corresponding author: Harshvardhan Chawla, Louisiana State University Health Sciences Center, 533 Bolivar Street (Suite 459), New Orleans, LA 70112.
